# The influence of age and weight on the outcomes of complete atrioventricular septal defect repair

**DOI:** 10.1186/s43044-022-00292-8

**Published:** 2022-07-18

**Authors:** Heidi K. Al-Wassia, Osman O. Al-Radi, Khadijah A. Maghrabi, Mawadda A. Bayazeed, Murooj M. Qattan, Doaa T. Ebraheem, Sarah U. Gadi, Mernan F. Kattan, Reema A. Alghamdi, Samaher H. Alzabidi, Ahmed M. Dohain

**Affiliations:** 1 Department of Pediatrics, College of Medicine, King Abdulaziz University, P.O. Box 80215, Jeddah, 21589 Saudi Arabia; 2 Cardiac Surgery Division, Department of Surgery, King Abdulaziz University, Jeddah, Saudi Arabia; 3 Pediatric Cardiology Division, Department of Pediatrics, King Abdulaziz University, Jeddah, Saudi Arabia; 4 Faculty of Medicine, King Abdulaziz University, Jeddah, Saudi Arabia; 5 Pediatric Cardiology Division, Department of Pediatrics, Cairo University, Cairo, Egypt; 6 Pediatric Cardiac Intensive Care Unit, Department of Pediatrics, King Abdulaziz University, Jeddah, Saudi Arabia

**Keywords:** Atrioventricular septal defect, Length of stay, Positive pressure ventilation, Age, Weight

## Abstract

**Background:**

The appropriate age and weight for surgical repair of atrioventricular septal defect (AVSD) is an area of controversy. We aimed to study the effect of weight and age at the time of surgical repair for complete AVSD in children less than 2 years of age on postoperative outcomes. A retrospective data review was performed for patients who underwent the AVSD repair from 2012 to 2019 at our institutions. Our primary outcome was the postoperative in-hospital length of stay (LOS). Secondary outcomes included total positive pressure ventilation (PPV), ventilation time, maximum vasoactive–inotropic score (max VIS), and other postoperative complications.

**Results:**

The study included fifty patients. The median age was 191 days, and the median weight was 4.38 kg at the time of surgery. Weight < 4 kg was associated with longer PPV time and postoperative in-hospital LOS (*p* value of 0.033 and 0.015, respectively). Additionally, they had higher max VIS at 24 h and 48 h than the other groups with bodyweight 4–5.9 kg or ≥ 6 kg (*p* value of 0.05 and 0.027, respectively). Patients with older age or lower weight at operation had a longer in-hospital LOS and total length of PPV after surgery. There were no postoperative in-hospital deaths.

**Conclusions:**

Older age and lower weight at the time of surgical repair of atrioventricular septal defect could be independent predictors of prolonged postoperative in-hospital length of stay and total length of positive pressure ventilation.

## Background

Atrioventricular septal defect (AVSD) is a spectrum of congenital heart disorders characterized by a deficiency of endocardial cushion tissue at the atrioventricular junction. An ostium primum atrial septal defect, a common atrioventricular valve, and a variable deficiency of the ventricular septum inflow typify the complete form. In the balanced complete AVSD type, the AV junction is connected unvaryingly to the right and left ventricles that receive a comparable amount of blood and are symmetrical in size. It is estimated that AVSD occurs in 4–5 out of every 10,000 live births, and it represents 4–5% of all congenital heart diseases (CHD) [1]. A hospital-based cross-sectional study in Saudi Arabia reported 36 AVSD cases out of 591 children (6%) with CHD [2]. Surgical repair to close the atrial and ventricular septal defects is usually performed in infancy. The appropriate age and weight at the time of surgery are controversial. A body of published evidence showed that infants with a weight less than 2.5 kg at the time of surgery for CHD had a higher rate of mortality and morbidity [3–6]. Conversely, several other studies asserted that delaying cardiac surgery to achieve weight gain is unwarranted and was not shown to be an independent risk factor for adverse postoperative outcomes [7, 8]. Determining whether weight and age at the time of operation for complete AVSD affect postoperative morbidity and mortality has not been studied in our region. Further, the findings from this study can guide decision-making that influences both operative and short- and long-term outcomes of infants undergoing cardiac surgery for complete AVSD.

We aimed to study the effect of weight and age at the time of surgical repair for complete AVSD in children less than 2 years of age on postoperative morbidity and mortality.

## Methods

### Population and settings

Approximately 260 congenital cardiac surgeries are performed annually in our center. The Pediatric Cardiac Center of Excellence at King Abdulaziz University Hospital is a tertiary and referral center in the Western Region of Saudi Arabia. Prospectively collected data were augmented with a retrospective chart review of all patients less than 2 years of age who underwent surgical repair for complete AVSD between January 1, 2012, and August 31, 2019. Patients who underwent previous cardiac surgery, unbalanced AVSD, and associated tetralogy of Fallot (TOF) or transposition of great arteries (TGA) were excluded. Patients with associated coarctation of the aorta (COA) or patent ductus arteriosus (PDA) who had those lesions repaired at the time of AVSD repair were included.

### Variables

Our primary outcome was postoperative in-hospital length of stay (LOS), defined as the time in days from operation till the time of discharge home. Our secondary outcomes included 1—total positive pressure ventilation (PPV) time, defined as the time to wean off all types of positive pressure ventilation, including endotracheal intubated mechanical ventilation and noninvasive positive pressure ventilation measured in days; 2—ventilation time (VT) is defined as the time in days from arrival to ICU after surgery to the first successful extubation and not requiring re-intubation within 24 h; 3—maximum vasoactive–inotropic score (Max VIS) which includes medications from the inotropic score (IS), a clinical tool used to quantify the need of inotropic cardiovascular support after cardiac surgery (IS = dopamine dose (μg/kg/min) + dobutamine dose (μg/kg/min) × 100 + epinephrine dose (μg/kg/min)) [9] in addition to medications that are typically used in contemporary clinical practice measured at 24 and 48 h after surgery (VIS = IS + 10 × milrinone dose (μg/kg/min) + 10,000 × vasopressin dose (Unit/kg/min) + 100 × norepinephrine dose\ (μg/kg/min)) [10]; 4—cardiopulmonary bypass (CPB) time in minutes, 5—aortic clamp time in minutes, 6—postoperative degree of valvular regurgitation based on subjective evaluation of the echocardiographic findings of the appearance and width of the color Doppler regurgitation jets of the right and left AV valves (RAVV, LAVV) at the time of discharge and classified as none\trace, mild, moderate, and severe [11]; 7—occurrence of heart block and whether it needed temporary or permanent pacemaker (TPM, PPM) implantation; 8—need for re-operation; 9—in-hospital mortality; 10—sepsis; 11—chylothorax; 12—supraventricular tachycardia requiring treatment; 13—need for extracorporeal membrane oxygenation (ECMO); and 14—need for dialysis.

The predictors of interest in the study were age and weight at the time of surgery measured in days and kilograms, respectively. Collected confounders included: gender, diagnosis of down syndrome, the urgent surgeries which included patients who underwent preoperative hospital admissions and could not be discharged from the hospital due to uncontrolled heart failure or the need for respiratory support, number of preoperative hospital admissions, preoperative length of stay (Preop LOS), defined as the time in days from hospital admission till the time of operation, total preoperative intensive care unit stay (Total Preop ICUS) defined as the number of ICU days in the preoperative period, need for preoperative positive pressure ventilation (Preop PPV) defined as continuous positive airway pressure (CPAP) or mechanical ventilation (MV), total preoperative positive pressure ventilation (Total Preop PPV) which is the number of preoperative days in which the patient required PPV, diagnosis of right/left ventricular hypoplasia, associated (COA) or patent ductus arteriosus (PDA), and preoperative intake of furosemide or captopril (mg/kg/day).

### Statistical analysis

R-project statistical package (www.r-project.org) was used for all statistical analyses. Continuous variables were described as median and interquartile ranges. Categorical variables were expressed as frequency and percentage. Differences between groups were examined using Fisher’s exact test for categorical data, Student’s t test for continuous normally distributed data, and Wilcoxon rank-sum calculations when the assumption of normality was violated. Multiple regression with quasi-Poisson distribution was used to assess the independent predictors of prolonged times (dependent variables). Weight, age, and length of PPV were included in the models as continuous variables. Normality and linearity were tested and adjusted where appropriate. Statistical significance was set at a *p* value of 0.05.

## Results

A total of fifty-eight consecutive patients underwent complete AVSD repair in the study period. Eight patients were excluded; two had previous COA repair and pulmonary artery banding, two had associated TOF repair, one had associated TGA, and three patients were older than 2 years of age. The remaining fifty patients constituted the study sample. The patients were divided into three weight groups for tabulation and data visualization. Fifteen patients were 2.4 to 3.9 kg, twenty-four patients were 4 to 5.9 kg, and eleven patients were ≥ 6 kg at the time of surgery. Patients in different weight categories were comparable in their preoperative medical condition except for the need and duration of preoperative respiratory support (Table 1). There were no postoperative in-hospital deaths. One patient died 1 year after surgery; his weight at the time of operation was 8.2 kg.

**Table 1:** Descriptive statistics by weight group

	*N*	Weight 2.4–3.9 kg *N* = 15	Weight 4.0–5.9 kg *N* = 24	Weight ≥ 6 kg *N* = 11	Combined *N* = 50	*p* value
Male	50	5 (33%)	7 (29%)	5 (45%)	17 (34%)	0.639
Age (days)	50	125 (92,192)	184 (152,233)	416 (260,461)	191 (148,253)	< 0.001
Weight (kg)	50	3.50 (2.95,3.70)	4.50 (4.20,5.10)	6.50 (6.30,7.30)	4.38 (3.80,5.68)	< 0.001
Weight (Z score)	50	− 5.49 (− 6.09, − 4.17)	− 4.21 (− 4.85, − 3.34)	− 2.80 (− 3.74, − 1.61)	− 4.17 (− 5.04, − 3.23)	< 0.001
Down syndrome	50	11 (73%)	20 (83%)	10 (91%)	41 (82%)	0.501
Urgent	50	10 (67%)	11 (46%)	3 (27%)	24 (48%)	0.133
Preop hospital admissions						
None	50	7 (47%)	15 (62%)	4 (36%)	26 (52%)	0.050
1		5 (33%)	8 (33%)	3 (27%)	16 (32%)	
2		3 (20%)	0 (0%)	1 (9%)	4 (8%)	
≥ 3		0 (0%)	1 (4%)	3 (27%)	4 (8%)	
Preop LOS (days)	50	16.0 (4.0,26.5)	1.0 (1.0,15.5)	1.0 (1.0,2.0)	2.0 (1.0,17.0)	0.074
Total preop ICUS (days)	50	2.0 (0.0,18.5)	1.0 (0.0,2.2)	1.0 (0.5,6.5)	1.0 (0.0,5.8)	0.405
Preop PPV						
CPAP	50	4 (27%)	1 (4%)	0 (0%)	5 (10%)	0.017
MV		5 (33%)	3 (12%)	1 (9%)	9 (18%)	
None		6 (40%)	20 (83%)	10 (91%)	36 (72%)	
Total preop PPV (days)	50	7.0 (0.0,15.5)	0.0 (0.0,0.0)	0.0 (0.0,0.0)	0.0 (0.0,0.8)	0.016
Preoperative LAVV regurgitation						
None, trace	43	5 (36%)	5 (26%)	4 (40%)	14 (33%)	0.827
Mild		5 (36%)	5 (26%)	1 (10%)	11 (26%)	
Moderate		3 (21%)	7 (37%)	4 (40%)	14 (33%)	
Severe		1 (7%)	2 (11%)	1 (10%)	4 (9%)	
Preoperative RAVV regurgitation						
None, trace	43	3 (21%)	2 (10%)	3 (30%)	8 (19)	0.643
Mild		3 (21%)	6 (32%)	2 (20%)	11 (25%)	
Moderate		6 (43%)	10 (53%)	5 (50%)	21 (49%)	
Severe		2 (14%)	1 (5%)	0 (0%)	3 (7%)	
LV hypoplasia	50	0 (0%)	1 (4%)	0 (0%)	1 (2%)	0.575
RV hypoplasia	50	0 (0%)	0 (0%)	1 (9%)	1 (2%)	0.164
Associated COA	50	1 (7%)	0 (0%)	0 (0%)	1 (2%)	0.304
Associated PDA	50	2 (13%)	5 (21%)	1 (9%)	8 (16%)	0.642
Preop furosemide	50	12 (80%)	21 (88%)	10 (91%)	43 (86%)	0.700
Furosemide dose (mg/kg/day)	50	2.76 (1.28,3.13)	2.56 (1.89,3.00)	1.67 (1.34,2.39)	2.42 (1.65,3.00)	0.431
Preop captopril	50	10 (67%)	18 (75%)	10 (91%)	38 (76%)	0.355
Captopril dose (mg/kg/day)	50	1.25 (0.00,1.79)	1.50 (0.33,2.14)	1.43 (1.08,2.13)	1.44 (0.36,2.10)	0.653

COA: coarctation of aorta, CPAP: continuous positive airway pressure, ICU: intensive care unit, LAVV: left atrioventricular valve, LV: left ventricle, LOS: length of stay, MV: mechanical ventilation, PDA: patent ductus arteriosus, Preop: preoperative, PPV: positive pressure ventilation, RAVV: right atrioventricular valve, RV: right ventricle

Using the Z score for weight, all included patients had low weight for age except 4 patients (92%), one patient had a body weight of 5.7 kg, and the other 3 patients had body weights ≥ 6 kg. Lower body weight for age was associated with longer postoperative in-hospital LOS, VT, and PPV time (*p* value was 0.05, 0.05, and 0.002, respectively). The group of patients with weight < 4 kg was associated with longer PPV time and postoperative in-hospital LOS (*p* value of 0.033 and 0.015, respectively). Additionally, they had higher max VIS at 24 h and 48 h compared to the other groups with bodyweight 4–5.9 kg or ≥ 6 kg (*p* value of 0.05 and 0.027, respectively) (Table 2). In patients less than 4 kg, one required a permanent pacemaker for complete heart block and two needed re-operation for valve repair.

**Table 2:** Outcomes by weight group

	Weight 2.4–3.9 kg *N* = 15	Weight 4.0–5.9 kg *N* = 24	Weight ≥ 6 kg *N* = 11	Combined *N* = 50	*p* value
Ventilation time (days)	3.00 (1.50,5.50)	1.50 (1.00,3.50)	1.00 (1.00,2.50)	2.00 (1.00,4.75)	0.156
Total PPV time (days)	11.00 (8.00,17.00)	7.00 (4.00,10.75)	5.00 (3.50,9.00)	8.00 (4.25,13.75)	0.033
CPB time (min)	75.00 (64.00,78.00)	75.50 (64.50,87.75)	81.00 (68.50,94.00)	75.50 (64.25,91.00)	0.726
Aortic clamp time (min)	58.00 (50.50,66.00)	62.50 (49.75,72.25)	59.00 (52.50,76.50)	60.00 (50.00,68.00)	0.771
Max VIS in 24 h	13.00 (10.00,15.00)	10.25 (7.88,15.00)	9.00 (2.50,13.75)	10.50 (8.25,15.00)	0.050
Max VIS in 48 h	15.00 (11.50,16.25)	11.75 (8.75,17.12)	9.00 (2.50,13.75)	12.50 (9.00,15.00)	0.027
Postoperative LOS (days)	17.00 (13.00,28.50)	9.00 (7.00,15.25)	10.00 (7.00,17.00)	12.50 (7.25,17.75)	0.015
Chylothorax	5 (33%)	1 (4%)	1 (9%)	7 (14%)	0.033
SVT requiring treatment	1 (7%)	0 (0%)	0 (0%)	1 (2%)	0.304
Heart block needed TPM	2 (13%)	1 (4%)	0 (0%)	3 (6%)	0.321
Heart block needed PPM	1 (7%)	0 (0%)	0 (0%)	1 (2%)	0.304
ECMO	1 (7%)	0 (0%)	0 (0%)	1 (2%)	0.304
Dialysis	0 (0%)	1 (4%)	0 (0%)	1 (2%)	0.575
Sepsis	5 (33%)	3 (12%)	1 (9%)	9 (18%)	0.176
Postoperative LAVV regurgitation					
None	11 (73%)	17 (71%)	11 (100%)	39 (78%)	0.324
Mild	4 (27%)	4 (17%)	0 (0%)	8 (16%)	
Moderate	0 (0%)	2 (8%)	0 (0%)	2 (4%)	
Severe	0 (0%)	1 (4%)	0 (0%)	1 (2%)	
Postoperative RAVV regurgitation					
None	13 (86%)	22 (92%)	11 (100%)	46 (92%)	0.620
Mild	1 (7%)	1 (4%)	0 (0%)	2 (4%)	
Moderate	1 (7%)	1 (4%)	0 (0%)	2 (4%)	
Severe	0 (0%)	0 (0%)	0 (0%)	0 (0%)	
Needed re-operation	2 (13%)	0 (0%)	0 (0%)	2 (4%)	0.088
In-hospital mortality	0 (0%)	0 (0%)	0 (0%)	0 (0%)	

CPB: cardiopulmonary bypass, ECMO: extracorporeal membrane oxygenation, LAVV: left atrioventricular valve, LOS: length of stay, Max: maximum, PPM: permanent pacemaker, PPV: positive pressure ventilation, RAVV: right atrioventricular valve, SVT: supraventricular tachycardia, TPM: temporary pacemaker VIS: vasoactive–inotropic score

**Fig. 1:**
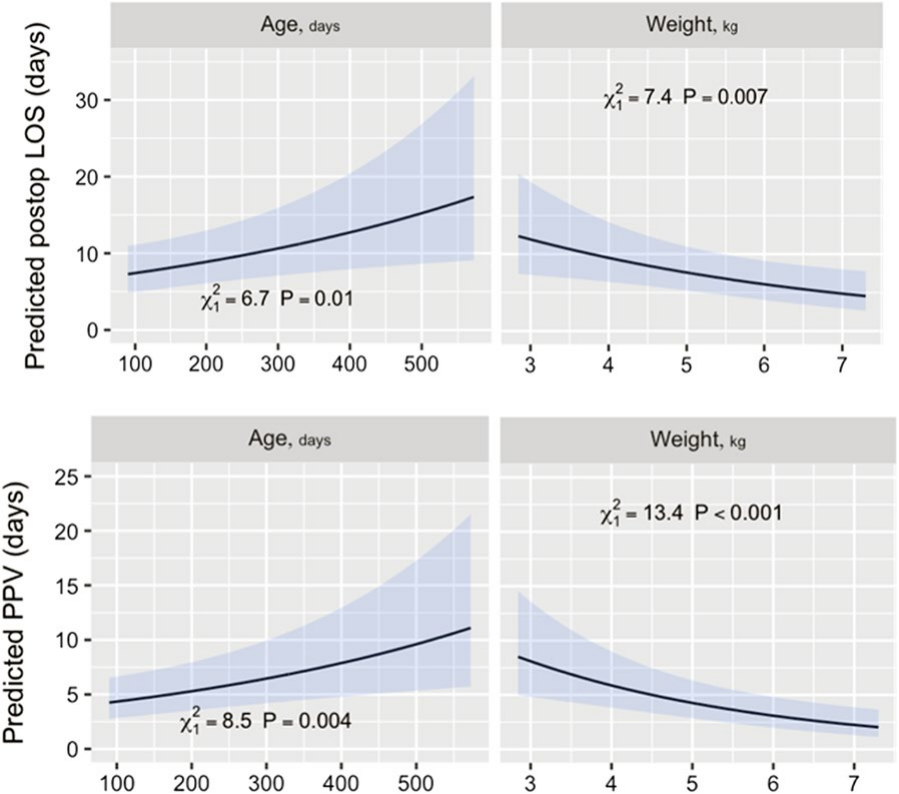
Predicted postoperative in-hospital length of stay (LOS) and positive pressure ventilation (PPV) for age and body weight

Measured at the time of surgery, older age, lower weight, male gender, and urgent surgery were independent predictors of prolonged postoperative in-hospital LOS (*p* values; 0.01, 0.007, 0.001, and 0.02, respectively) (Table 3, Fig. 1). The effects of age and weight were in opposite directions. Patients with younger age at operation had a shorter postoperative in-hospital LOS after surgery. For a 100-day-old patient, the predicted LOS was 8 days, whereas, for a 400-day-old patient, it was 12 days (Fig. 1). On the other hand, patients with lower weight had a longer predicted postoperative in-hospital LOS. A 3-kg baby is predicted to stay 12 days in the hospital, whereas a 5-kg baby will stay 8 days postoperatively (Fig. 1). The same factors were also predictive of total length of PPV (*p* values; 0.004, 0.001, 0.001, and 0.03, respectively) (Table 3, Fig. 1). Again, the effects of age and weight were in opposite directions. A 100-day-old baby is predicted to require PPV for 4 days, whereas a 400-day-old baby is predicted to require it for 8 days. On the other hand, a 3-kg baby is predicted to require PPV for 7 days, versus a 5-kg baby is predicted to require it for 4 days (Fig. 1). The presence of down syndrome was not associated with increased postoperative in-hospital LOS (*p* = 0.3), but it was associated with longer VT and total PPV time; the *p* value was 0.0003 and 0.041, respectively (Table 3).

**Table 3:** Multivariable predictors of outcomes

	Postoperative in-hospital LOS			Ventilation time			Total PPV time		
	Coef	SE	*p* value	Coef	SE	*p*	Coef	SE	*p*
Intercept	3.0629	0.388	< 0.0001	3.0152	0.708	< 0.0001	3.1634	0.405	< 0.0001
Male gender	0.6404	0.189	0.001	0.8211	0.321	0.011	0.6793	0.202	0.001
Age (days)	0.0018	0.001	0.010	− 0.0010	0.001	0.460	0.0020	0.0007	0.004
Weight (kg)	− 0.2241	0.081	0.007	− 0.2756	0.161	0.086	− 0.3209	0.088	0.0003
Down syndrome	− 0.2570	0.235	0.300	− 1.2529	0.350	0.0003	− 0.4891	0.240	0.041
Urgent	0.5021	0.211	0.018	0.6234	0.348	0.073	0.4875	0.224	0.030
Preoperative PPV (days)	0.0019	0.006	0.800	− 0.0017	0.011	0.900	0.0098	0.006	0.096

Coef, coefficient; LOS, length of stay; PPV, positive pressure ventilation; SE, standard error

## Discussion

We studied fifty patients under 2 years of age who underwent surgical repair for complete AVSD. In our sample, older age and lower weight at the time of surgery significantly predicted a more extended period of postoperative PPV and hospital stay. The patients with the shortest hospital stay and need for PPV were the ones who were young but gaining good weight. Therefore, delaying surgery in hopes of better outcomes is only valid if the baby gains weight.

Recent studies reported improved postoperative survival over the years, attributed to enhanced factors related to early diagnosis, preoperative management, surgical techniques, intraoperative support, and postoperative care [12, 13].

Alsoufi et al. examined the effect of weight below 2.5 kg on surgical outcomes in infants less than 3 months who underwent congenital cardiac surgery. Contrary to our study, the author demonstrated a significant association between lower weight and prolonged postoperative VT and ICU and total hospital stay [6]. However, those who weighed less than 2.5 kg in the same study had a significantly higher incidence of prematurity and chromosomal and extracardiac anomalies. Similarly, in a large multicenter cohort of 2399 patients who underwent complete AVSD repair over 4-year period, weight < 3.5 kg and age ≤ 2.5 months were associated with higher rates of mortality and postoperative LOS [14]. In our study that was not the case, we had no operative mortality in any of the age or weight groups. In the same study, infants ≤ 2.5 months had a higher prevalence of preoperative ventilatory and circulatory support. There was no report on the degree of PAVV regurgitation or other preoperative description of AVV morphology associated with unfavorable outcomes [14]. In an earlier multicenter study by Atz et al., which included 120 children with complete AVSD who underwent surgical repair, adverse effects were negatively correlated with the repair at < 2.5 months but did not differ by age past 2.5 months. In the same study, the younger age-group had more repairs using the Australian technique and were more likely to endure circulatory arrest [15]. A recent single-center retrospective study of 153 patients with complete AVSD without associated cardiac abnormalities reported increased mortality, length of hospital stay, and re-operation within 1 year of primary repair with a more minor younger age in the bivariate analysis. However, the association was only significant for more extended hospital stay after adjustment for other covariates. In the same study, 33% of the patients were born prematurely less than 37 weeks, and prematurity was an independent risk factor of the studied outcomes. Moreover, the birth weight of the included patients ranged from 1.2 to 4.39, and weight was not entered in the multivariate regression model because of collinearity with other variables [16]. There has been an argument against delaying congenital cardiac surgery awaiting weight gain as that might result in increased severity and irreversibility of the associated pulmonary hypertension and the consequent congestive heart failure, which are not always controlled by anti-failure medications. Moreover, patients can develop recurrent chest infections waiting for repair, further exacerbating abnormal pulmonary vascular resistance. Delaying operation can also be a potential mechanism of progressive degenerative changes of the AV valve, preoperative AVVR, and deterioration of ventricular dilation, the severity of which was found to increase the risk of postoperative morbidities and re-operation [12, 13, 17]. Consistent with these reports, we found that low weight was associated with longer PPV time, prolonged postoperative in-hospital LOS, and higher max VIS at 24 and 48 h. Moreover, the two patients who required re-operation had low weight for age at primary surgical repair. However, there was no significant difference in the severity of AVV regurgitation in different weight groups pre- or postoperatively.

Re-operation for AVVR continues to be a significant problem after complete AVSD repair. The reintervention rates reported in other studies range between 10 and 20% at 10 years [13, 18].

Like other reports, the diagnosis of trisomy 21 was not associated with an increased risk of postoperative in-hospital LOS [13–15]. Down syndrome patients are more likely to have the balanced form of AVSD and less likely to have heterotaxy syndrome, which might explain the better surgical outcomes [19]. Moreover, patients with normal chromosomes are expected to have a greater degree of left to right shunt and are more likely to have growth failure at the time of surgery than those with trisomy 21 [15].

We want to acknowledge the strengths and limitations of this study. The strengths of this study included the detailed pre- and postoperative recorded clinical information about the included patients that were harvested and utilized prospectively in a single center, which will lessen the effect of information bias. The use of age and weight as continuous variables in the regression model that included other confounders was a strength of the analysis, as the inverse effect of weight and age changes on the postoperative morbidities was made clear. Due to the study's retrospective nature, the gestational age and birth weight were not available for most of the patients referred from other hospitals, which could provide more details about the weight gain of those patients.

Limitations of this study included referral bias, as our center is a tertiary and referral center for infants with congenital cardiac diseases that need intervention or surgery. Center variability in preoperative medical management and timing of surgical referral may introduce referral bias affecting the outcome of surgical intervention. In addition, variation in surgical technique and approach may predict outcomes, and in our center, our cohort was operated by a single surgeon, which might not reflect the variability in practice and make the results of this study generalizable.

## Conclusions

Older age, lower weight, male gender, and urgent surgery could be independent predictors of prolonged postoperative in-hospital length of stay and total length of positive pressure ventilation for children undergoing atrioventricular septal defect surgical repair.

## Data Availability

The datasets used and/or analyzed during the current study are available from the corresponding author on reasonable request.

## Abbreviations

AVSD: Atrioventricular septal defect
CHD: Congenital heart disease
COA: Coarctation of the aorta
CPAP: Continuous positive airway pressure
CPB: Cardiopulmonary bypass
ECMO: Extracorporeal membrane oxygenation
IS: Inotropic score
LAAV: Left atrioventricular valve
LOS: Length of stay
MV: Mechanical ventilation
PDA: Patent ductus arteriosus
PPM: Permanent pacemaker
PPV: Positive pressure ventilation
RAVV: Right atrioventricular valve
TGA: Transposition of great arteries
TOF: Tetralogy of Fallot
TPM: Temporary pacemaker
VIS: Vasoactive–inotropic score
VT: Ventilation time
